# Supplementary material for: Barley Leaf Insoluble Dietary Fiber Alleviated Dextran Sulfate Sodium-Induced Mice Colitis by Modulating Gut Microbiota
Source: Nutrients. 2021 Mar 5;13(3):846. doi: 10.3390/nu13030846 (PMC8001343; doi:10.3390/nu13030846)
Supplement: Supplementary file 1 [file nutrients-13-00846-s001.pdf]

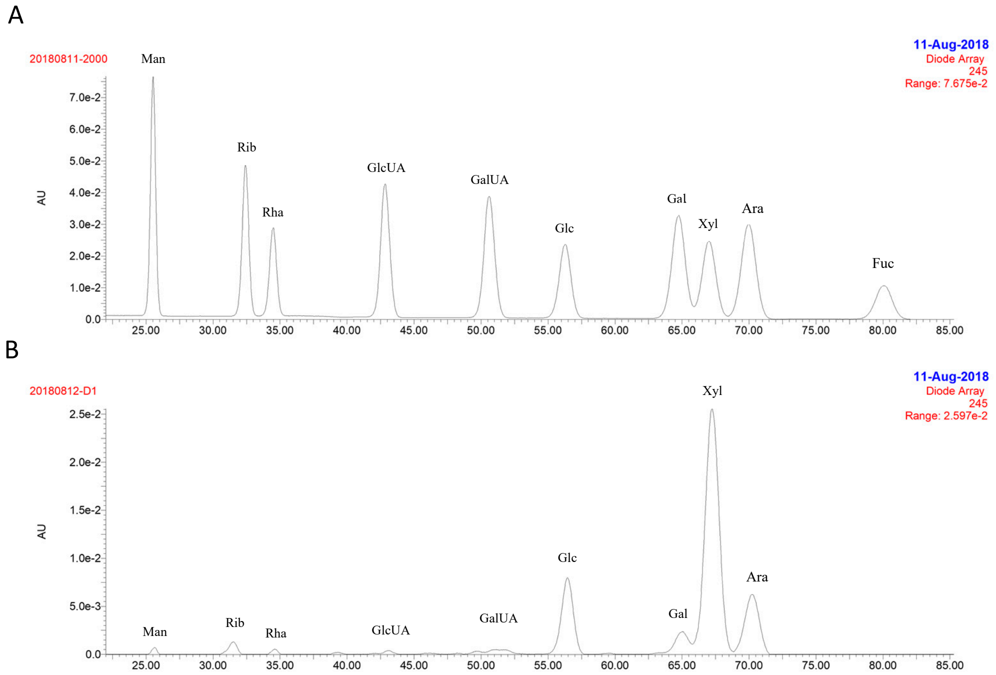

Supplementary Figure S1. The HPLC chromatograms of monosaccharide standards (**A**) and monosaccharides released from barley leaf insoluble dietary fiber (BLIDF) (**B**); Peaks: Man, mannose; Rib, ribose; Rha, rhamnose; GlcUA, glucuronic acid; GalUA, galacturonic acid; Glc, glucose; Gal, galactose; Xyl, xylose; Ara, arabinose; Fuc, Fucose.

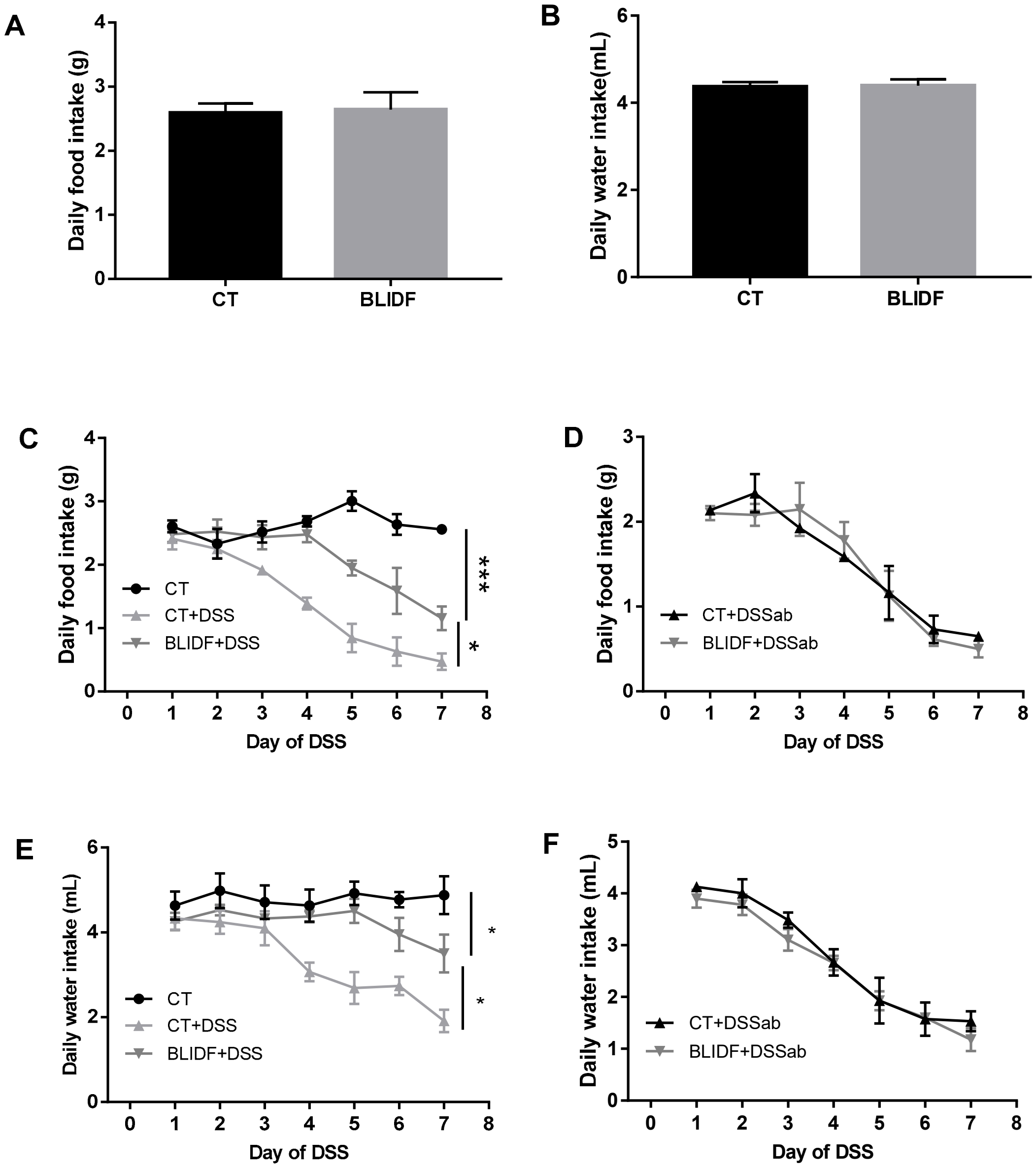

Supplementary Figure S2. Daily food intake (**A**) and water intake (**B**) in mice fed control and BLIDF diet under normal condition. Mouse food intake in SPF mice (**C**) and antibiotic-treated mice (**D**) under DSS administration. Mouse water intake in SPF mice (**E**) and antibiotic-treated mice (**F**) under DSS administration. * $p < 0.05$, ** $p < 0.01$, *** $p < 0.001$.

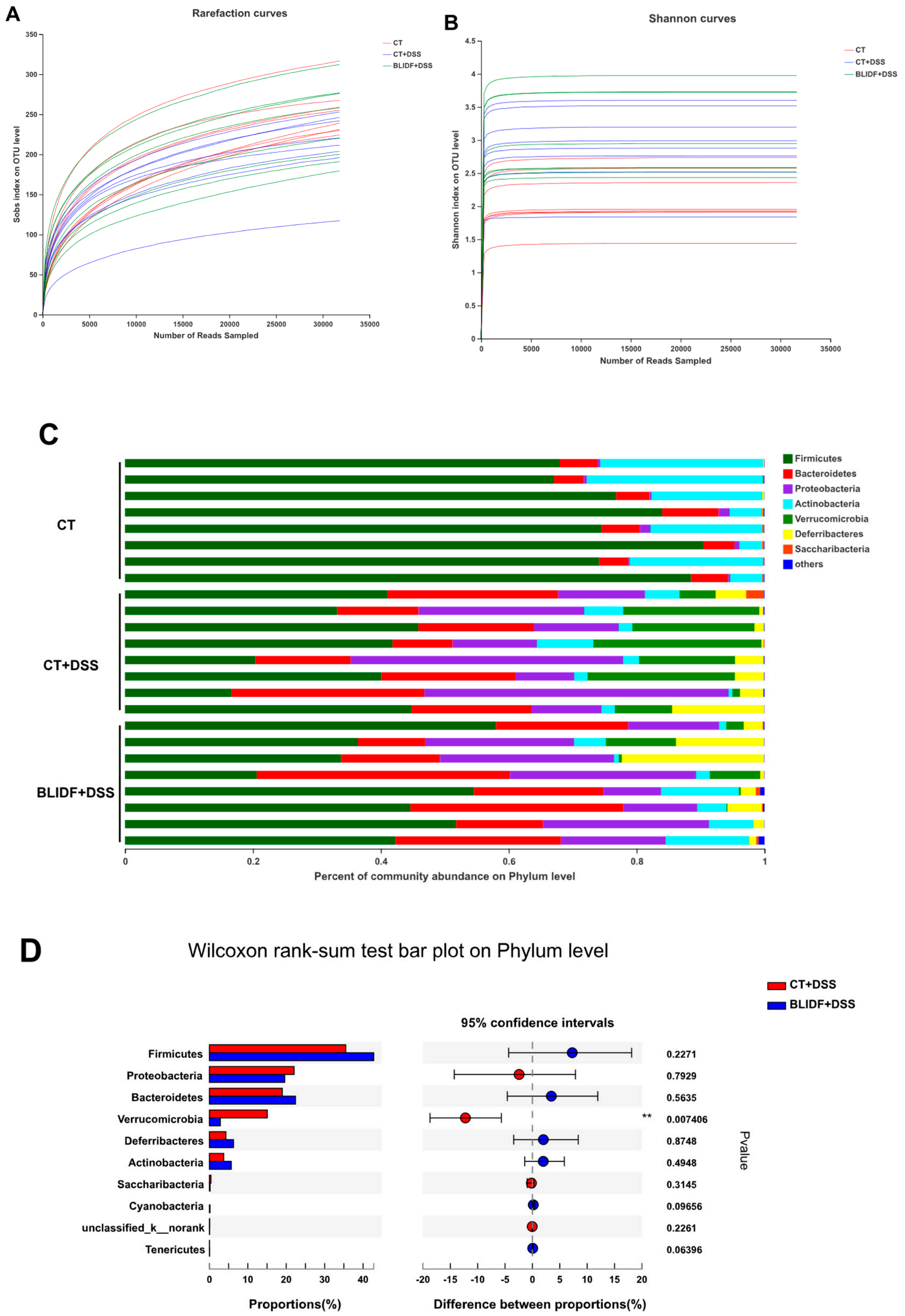

A
Rarefaction curves
Sobs index on OTU level
Number of Reads Sampled
CT
CT+DSS
BLIDF+DSS
B
Shannon curves
Shannon index on OTU level
Number of Reads Sampled
C
CT
CT+DSS
BLIDF+DSS
Firmicutes
Bacteroidetes
Proteobacteria
Actinobacteria
Verrucomicrobia
Deferribacteres
Saccharibacteria
others
Percent of community abundance on Phylum level
D
Wilcoxon rank-sum test bar plot on Phylum level
CT+DSS
BLIDF+DSS
95% confidence intervals
Firmicutes 0.2271
Proteobacteria 0.7929
Bacteroidetes 0.5635
Verrucomicrobia ** 0.007406
Deferribacteres 0.8748
Actinobacteria 0.4948
Saccharibacteria 0.3145
Cyanobacteria 0.09656
unclassified_k__norank 0.2261
Tenericutes 0.06396
Pvalue
Proportions(%)
Difference between proportions(%)

E

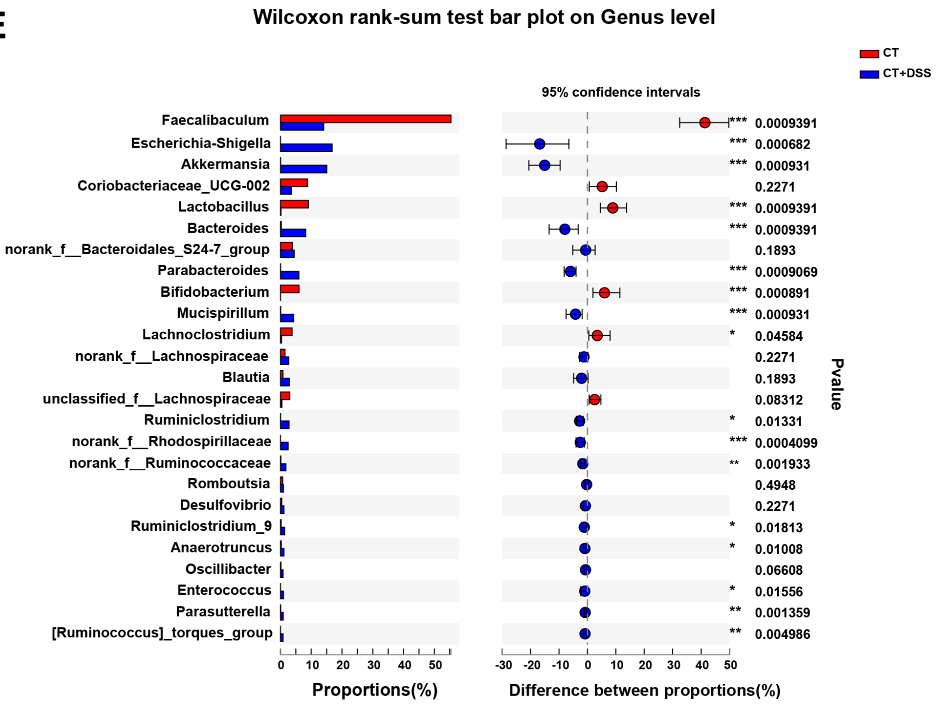

F

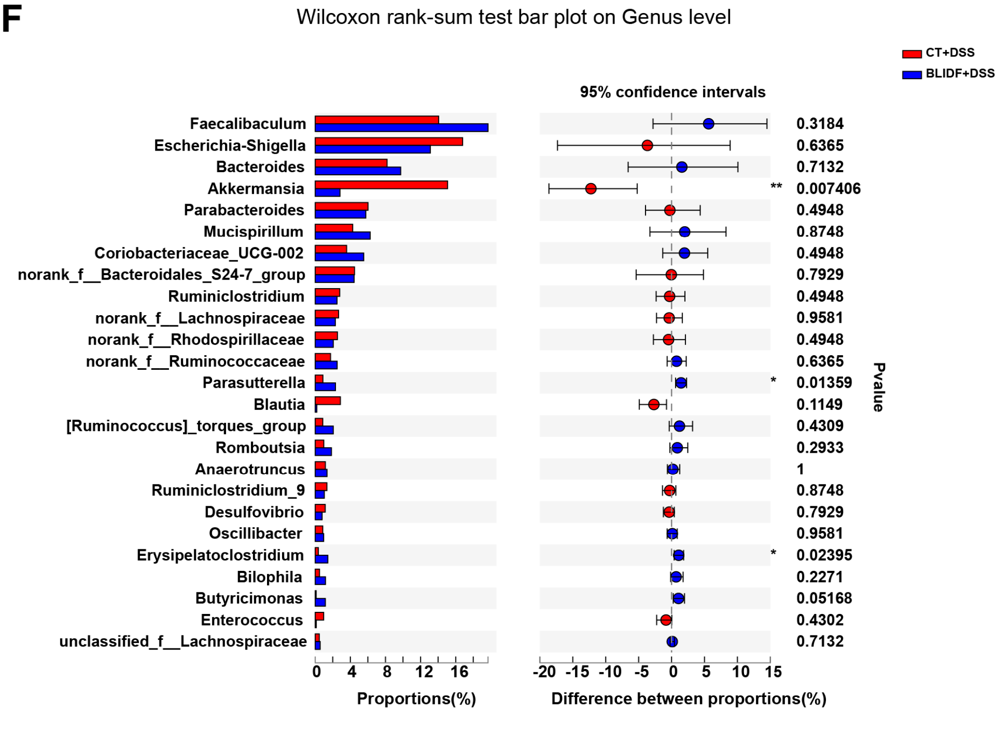

Supplementary Figure S3. (**A**) Rarefaction curves; (**B**) Shannon–Wiener curves; (**C**) Gut bacterial composition at the phylum level; (**D**) Comparison of gut bacterial composition of CT+DSS and BLIDF+DSS groups at the phylum level (determined Wilcoxon rank-sum test at FDR < 0.05); Comparison of gut bacterial composition of CT and CT+DSS groups (**E**) and CT+DSS and BLIDF+DSS groups (**F**) at the genus level (determined Wilcoxon rank-sum test at FDR < 0.05); n = 8 per group, * $p < 0.05$, ** $p < 0.01$, *** $p < 0.001$.

Supplementary Table S1. Nutrient contents of BLIDF.

	g/100g BLIDF
Protein	5.02 ± 0.10
Fat	0 ± 0
Moisture	4.12 ± 0.17
Cellulose	47.01 ± 1.16
Hemicellulose	25.52 ± 0.69
Lignin	17.16 ± 0.26
Acid soluble lignin	5.76 ± 0.18
Acid insoluble lignin	11.40 ± 0.16

Data were expressed by means ± SEM (n=3) and were expressed as “g/100 g dry basis”.

Supplementary Table S2. The composition of control diet and BLIDF diet.

Ingredients (g/kg of diet)	Diets	
	Control diet	BLIDF diet
Barley leaf flour	0	15.2
Casein	189.58	189.58
L-Cysteine	2.84	2.84
Corn Starch	298.59	298.59
Maltodextrin	33.18	33.18
Sucrose	331.77	331.77
Cellulose	47.40	47.40
Soybean oil	23.70	23.70
Lard	18.96	18.96
Mineral Mix M1002	9.48	9.48
Dicalcium Phosphate	12.32	12.32
Calcium Carbonate	5.21	5.21
Potassium Citrate	15.64	15.64
Vitamin mix V10001	9.48	9.48
Choline Bitartrate	1.90	1.90
Total	1000	1015.2

Supplementary Table S3. Primer sequences for qRT-PCR.

Gene	Amplicon size (bp)	Sequences (Forward/Reverse 5'-3')	reference
Occludin	129	TTGAAAGTCCACCTCCTTACAGA<br>CCGGATAAAAAGAGTACGCTGG	[1]
Claudin-1	204	AGGTCTGGCGACATTAGTGG<br>CGTGGTGTTGGGTAAGAGGT	[2]
Claudin-3	183	GAGATGGGAGCTGGGTTGTA<br>GTAGTCCTTGCGGTCGTAGG	[2]
Mucin2	282	CTGACCAAGAGCGAACACAA<br>CATGACTGGAAGCAACTGGA	[3]
GAPDH	248	GTGTTCCTACCCCCAATGTGT<br>ATTGTCATACCAGGAAATGAGCTT	[4]

1. Zhong, W.; Zhao, Y.T.; McClain, C.J.; Kang, Y.J.; Zhou, Z.X. Inactivation of hepatocyte nuclear factor-4 alpha mediates alcohol-induced downregulation of intestinal tight junction proteins. *Am J Physiol-Gastr L* **2010**, *299*, G643-G651, doi:10.1152/ajpgi.00515.2009.
2. Chen, G.X.; Huang, B.X.; Fu, S.P.; Li, B.; Ran, X.; He, D.W.; Jiang, L.Q.; Li, Y.H.; Liu, B.D.; Xie, L.W., et al. G Protein-Coupled Receptor 109A and Host Microbiota Modulate Intestinal Epithelial Integrity During Sepsis. *Front Immunol* **2018**, *9*, 1-12, doi:ARTN 2079 10.3389/fimmu.2018.02079.
3. Zarepour, M.; Bhullar, K.; Montero, M.; Ma, C.X.; Huang, T.; Velcich, A.; Xia, L.J.; Vallance, B.A. The Mucin Muc2 Limits Pathogen Burdens and Epithelial Barrier Dysfunction during Salmonella enterica Serovar Typhimurium Colitis. *Infect Immun* **2013**, *81*, 3672-3683, doi:10.1128/Iai.00854-13.
4. Li, J.; Ying, H.C.; Cai, G.Y.; Guo, Q.; Chen, L.Z. Impaired proliferation of pancreatic beta cells, by reduced placental growth factor in pre-eclampsia, as a cause for gestational diabetes mellitus. *Cell Proliferat* **2015**, *48*, 166-174, doi:10.1111/cpr.12164.
